# Medical management of an ovarian ectopic pregnancy: a case report

**DOI:** 10.1186/s13256-015-0774-6

**Published:** 2015-12-20

**Authors:** Ozer Birge, Mustafa Melih Erkan, Ertugrul Gazi Ozbey, Deniz Arslan

**Affiliations:** 1 Nyala Sudan Turkey Training and Research Hospital, Department of Gynecology and Obstetrics, West Alezza District, Southern Nyala, Darfur Sudan; 2 Celal Bayar University Hospital, Department of Gynecology and Obstetrics, Manisa, Turkey; 3 Nyala Sudan Turkey Training and Research Hospital, Department of Urology, Nyala, Darfur Sudan

**Keywords:** Ectopic pregnancy, Methotrexate, Ovarian pregnancy

## Abstract

**Background:**

Primary ovarian ectopic pregnancy is a rare type of ectopic pregnancy which has an estimated prevalence ranging from 1:7000 to 1:70,000 accounting for almost 3 % of all ectopic cases. Here we report the case of a 25-year-old woman who presented to our clinic with abdominal pain, 6 weeks’ delay of menstruation and 3 days of vaginal bleeding, whose transvaginal ultrasonography showed an ectopic gestational sac with yolk sac inside, in her right ovary. This case shows that early diagnosis is very important particularly in places like the Sub-Saharan region of Africa.

**Case presentation:**

A 25-year-old African woman was referred to our clinic with 6 weeks’ delay of menstruation, frequent increasing abdominal pain and 3 days of vaginal bleeding. Her general condition was good and her vital signs were normal. She felt tenderness in an abdominal examination and had a small amount of vaginal bleeding. Transvaginal ultrasonography showed an ectopic gestational sac with yolk sac inside, in her right ovary. Our final diagnosis was ectopic ovarian pregnancy and we successfully treated her with methotrexate. After 3 weeks of methotrexate administration her beta human chorionic gonadotropin was negative and a sonographic examination was completely normal.

**Conclusions:**

Ectopic ovarian pregnancy is a very important medical situation. It should be diagnosed in its early stages otherwise it could be life-threatening and surgical treatment may be inevitable. Because of the importance of fertility, medical treatment is an acceptable option and can be feasible with early diagnosis.

## Background

Primary ovarian ectopic pregnancy is a rare type of ectopic pregnancy which has an estimated prevalence ranging from 1:7000 to 1:70,000 accounting for almost 3 % of all ectopic cases [1]. It is usually terminated by a rupture in the first trimester and because of the increased vascularization of the ovarian tissue it leads to internal hemorrhage and hypovolemic shock status. The diagnosis is usually made by emergency laparotomies and histopathologic assessment.

Diagnosis is made using the Spiegelberg criteria [2] which include:

- The gestational sac is located in the region of the ovary.
- The ectopic pregnancy is attached to the uterus by the ovarian ligament.
- Ovarian tissue in the wall of the gestational sac is proved histologically.
- The tube on the involved side is intact.

Non-tubal pregnancies are the most common type of ectopic pregnancy and ovarian pregnancies are the second most common type; ovarian pregnancies are very common with intrauterine devices (IUDs). Surgical treatments are often performed in these cases because of the late onset of clinical symptoms which leads to late diagnosis [1, 2]. Methotrexate (MTX) treatment can be used for patients in the early phases if their condition is stable.

## Case presentation

**Fig. 1:**
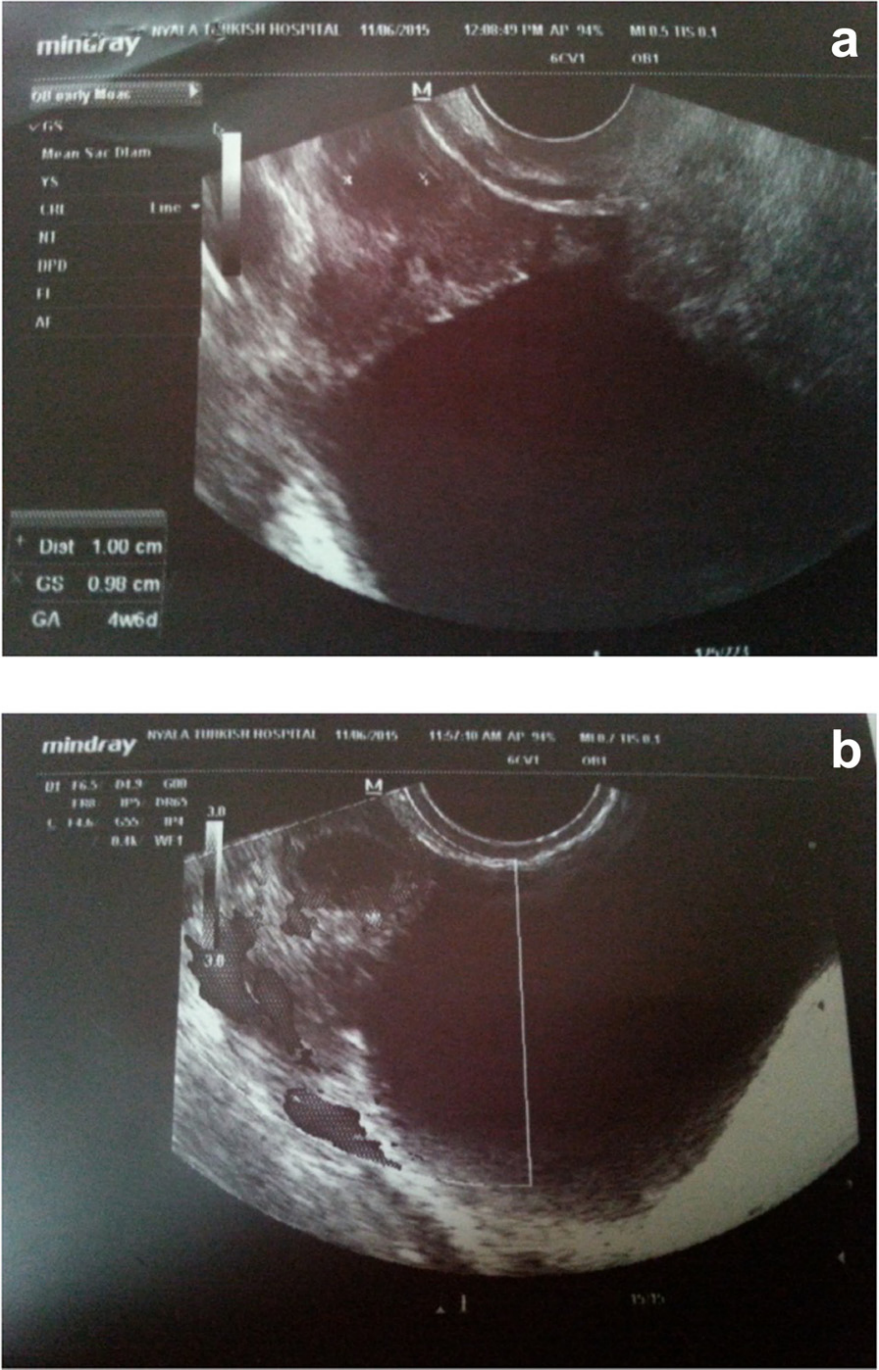
Ultrasound images of ovarian gestational sac before medical methotrexate therapy. (**a**) Ovarian gestational sac (**b**) Colour doppler image of gestational sac

**Fig. 2:**
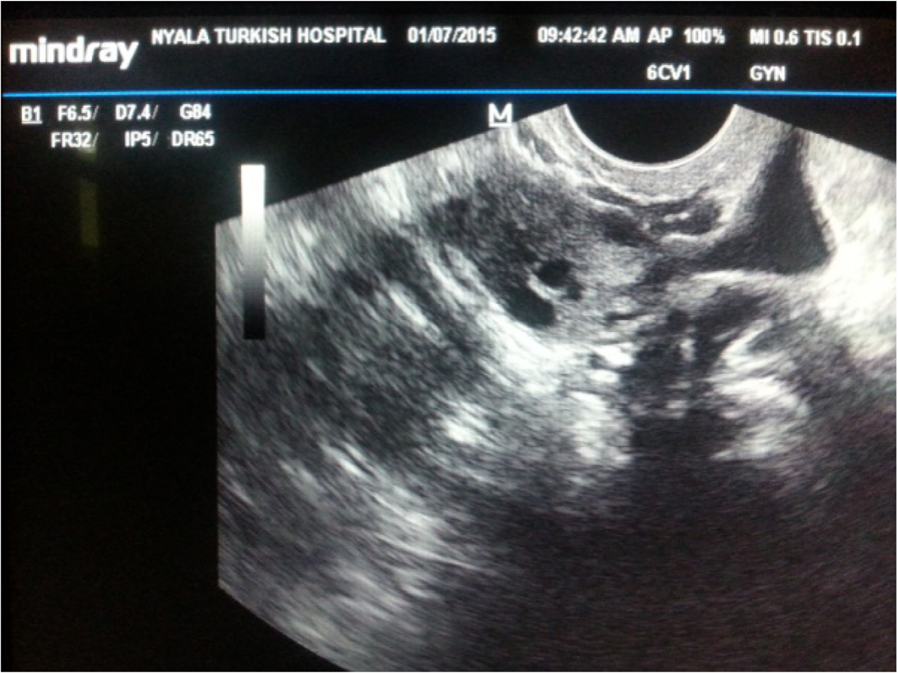
Control ultrasonographic image after the methotrexate treatment

A 25-year-old African obese woman with a history of two cesarean sections was referred to our clinic with 6 weeks’ delay of menstruation, frequent increasing abdominal pain and 3 days of vaginal bleeding. She had a regular menstrual period before the symptoms. In her medical history there was no record of use of an IUD, endometriosis or pelvic inflammatory disease. This was her third spontaneous pregnancy and there was no abortion. Her general condition was good and her vital signs were normal: blood pressure 110/70 mmHg, pulse 70 beats per minute (bpm), temperature 36.5 °C. A physical examination showed minimal tenderness in all sides of her abdomen with an increase in right lower pelvic section. A speculum examination showed a small amount of cervical bleeding, a palpable mass in rectouterine cavity and increased temperature and tenderness at right adnexial region. Transvaginal ultrasonography (USG) showed empty uterine cavity with 11 mm thickness. However, her rectouterine cavity was observed to be filled with heterogenous liquid including septations and hyperechogenic areas which were thought to be a coagulum. An ectopic gestational sac and yolk sac seemed to be inside her right ovary, and were identified close to the midline, which correlated with her 6 weeks’ delay of menstruation (Fig. 1). The fetus and fetal heart beat were not clearly seen. Vascular proliferation called ‘ring of fire’ which is typical for ectopic ovarian pregnancy was detected around the gestational sac (Fig. 1). Her left ovary and tubal structures seemed to be normal. She declared her previous menstrual periods were regular but that her last period was 2 months ago. Laboratory analysis showed a white blood cell count (WBC) of 11,600/mm^3^, red blood cell count (RBC) of 400000/mm^3^, hemoglobin (Hb) of 12.3 g/dl, hematocrit (Htc) of 36 %, beta human chorionic gonadotropin (HCG) of 6580 and normal urine results. She was diagnosed as having an ectopic ovarian pregnancy and was hospitalized. She and her family were informed about the stability of the condition and in view of her history of two previous cesarean sections, medical treatment of MTX was planned. A single dose of 90 mg intramuscular MTX was administered. She was stable. A progressive decrease in her beta HCG levels (4310 at fourth day, 2190 at seventh day, 210 at 14th day) as well as a diminishing of intraabdominal liquid and significant regression of her right ovarian sac were observed and she was discharged with weekly beta HCG test control advice. At the third week after the MTX treatment her beta HCG level was below 5 and her intraabdominal fluid had nearly disappeared (Fig. 2).

## Discussion

The history of ectopic pregnancy is as old as humanity. The first successful operation for ectopic pregnancy took place in 1759 in the USA but the usual treatment was still medical up to the 1800s with a maternal mortality rate reaching up to 60 % [3]. The high mortality rates drew special attention which led to crucial developments in the diagnosis and treatment of this condition. Salpingectomy, which started to be performed from the 1800s, is observed to be lifesaving because it decreased the maternal mortality rates to nearly 5 %.

Ovarian ectopic pregnancy is a rare variant of ectopic pregnancy [4]. It occurs by fertilization of an ovum retained in the peritoneal cavity leading to implantation on the ovarian surface [5]. Women with ovarian ectopic pregnancies usually present with lower abdominal pain, menstrual irregularities as in other ectopic conditions and corpus luteum cyst. Although early diagnosis and early treatment are crucial, preoperative and sometimes intraoperative diagnoses are difficult. Diagnosis is usually made by pathological assessment and therefore the Spiegelberg criteria are very important for the diagnosis of ectopic ovarian pregnancy [6].

Prediagnosis is usually supported by increased beta HCG levels. The current data inform that most cases occur in the first trimester. Early onset rupture can lead to massive intraabdominal hemorrhage resulting in hypovolemia which can be life-threatening. Some rare cases that reach second trimester are also documented [7]. There are also published cases of twin ovarian ectopic pregnancies and coincidence of uterine and ovarian ectopic pregnancies [7–10]. We also found some articles on ectopic pregnancies of advanced gestational age diagnosed preoperatively with USG and magnetic resonance imaging (MRI) [7, 11]. In the study of Hallat, a preoperative diagnosis was achieved in 28 % of 25 primary ectopic pregnancy cases. All other cases were diagnosed by pathological assessment postoperatively [12]. Phupong and Ultchaswadi declared that the evaluation of beta HCG together with transvaginal USG can be helpful for early diagnosis [13].

The cause of implantation anomalies in ovarian ectopic pregnancy is not clear [7, 12, 13]. There are various hypotheses such as:

- Delay of ovum liberation.
- Thickening of tunica albuginea.
- Tubal dysfunction.
- Intrauterine contraception devices (for example, IUDs).

Pelvic inflammatory disease does not have an effect on ovarian ectopic pregnancy like it does on tubal pregnancy [9, 14]. IUDs are thought to be a main factor in ovarian ectopic pregnancy cases according to the majority of studies. It is believed that IUDs trigger mild inflammation that disturbs the ciliary activity of the endosalpinx and leads to ovum transport delay and ectopic implantation [15, 16]. In our case, ectopic pregnancy was diagnosed from clinical and laboratory examinations and evaluations of her condition. Because of her two previous cesarean sections and the suspicion of secondary salpingitis by endemic chronic pelvic infections we performed medical treatment with MTX.

Primary ovarian ectopic pregnancy is usually seen among young fertile multipara women who use an IUD [17]. Berger and Blechner documented that the ratio of ovarian ectopic pregnancy among women using an IUD to all ectopic cases is 1:9; its prevalence in the general population is detected as 1:150 to 200 [16]. Our case had no history of IUD usage. In the case series of Raziel *et al*., 18 of 20 cases of ovarian pregnancy were using an IUD [14]. The link between IUDs and ovarian pregnancy in fertile patients is worthy of comment. In their study, Lehfeldt *et al*. detected that the IUDs prevent uterine implantation by 99.5 % and tubal implantation by 95.5 %; however, there is no preventive effect on ovarian implantation [18].

As the definitive diagnosis is made surgically and histopathologically even in patients with early onset, surgical interventions have both a diagnostic and a therapeutic value. Because oophorectomy is a radical procedure for ovarian ectopic pregnancy, consideration should be given to the patient’s age, fertility, her desire to have further pregnancies, and the size of the mass; wedge resection can also be another surgical option.

Medical and conservative treatments have also been introduced in recent years to prevent ovarian tissue loss, pelvic adhesions and to preserve the patient’s fertility. These include administration of mifepristone for patients diagnosed using a transvaginal USG, parenteral prostaglandin F2a and MTX treatment for non-ruptured cases detected with laparoscopy [11, 19]. Pagidas and Frishman performed MTX treatment for ovarian ectopic cases diagnosed using transvaginal USG and achieved healing. They emphasized that early staged cases diagnosed by transvaginal USG, can benefit from MTX treatment [20]. Di Luigi *et al.* also performed and succeeded with multidose MTX treatment which they administered to a 37-year-old patient with a history of two previous cesarean sections and IUD usage; she was diagnosed at 6 weeks of ectopic ovarian pregnancy by use of a transvaginal USG. They emphasized that with careful clinical evaluation and transvaginal examination early staged ovarian ectopic cases can be treated medically which preserves the normal anatomy crucial for fertility [21]. A review of the data shows that MTX treatment is chosen after a clear diagnosis and detection of the localization of ectopic cases by laparoscopy and therefore laparoscopy is declared to be a supporting diagnostic procedure [22]. In cases in which the gestational sac is lower than 30 mm, without fetal cardiac activity, and less than 6-weeks old, MTX treatment is supported in particular and is superior to surgery because it does not disturb fertility [23].

In our case although she had pelvic fluid of hemorrhagic character that could have been caused by pelvic rupture, a clinical evaluation and consideration of her previous operations led us to treat her medically. Her beta HCG levels progressively decreased after single dose MTX and she did not face the risks of further surgery.

## Conclusions

Although ovarian ectopic pregnancy is a rare condition, after careful evaluation, the selection of medical procedures should take into consideration the preservation of fertility particularly for young patients.

## Consent

Written informed consent was obtained from the patient for publication of this case report and any accompanying images. A copy of the written consent is available for review by the Editor-in-Chief of this journal.

## Abbreviations

HCG: Human chorionic gonadotropin
IUD: Intrauterine device
MTX: Methotrexate
USG: Ultrasonography

## References

[1] Marcus SM, Brinsden PR. Primary ovarian pregnancy after *in vitro* fertilization and embryo transfer: report of seven cases. Fertil Steril. 1993;60:167–70. 8513937

[2] Gerin-Lajoie L. Ovarian pegnancy. Am J Obstet Gynecol. 1951;62:920–9. 10.1016/0002-9378(51)90179-2 14885275

[3] Diamond MP, DeCherny AH. Ectopic pregnancy. WB Saunders. 1991;163:780–804.

[4] Al-Meshari AA, Chowdhury N, Adelusi BX. Ovarian pregnancy. Int J Gynaecol Obstet. 1993;41(3):269–72. 10.1016/0020-7292(93)90555-b 8102987

[5] Sturm JT, Hankins DG, Malo JW, Cicero JJ. Ovarian ectopic pregnancy. Ann Emerg Med. 1984;13:362–4. 10.1016/s0196-0644(84)80121-3 6711934

[6] Spiegelberg OX. Zur casuistic der ovarial schwangerschaft. Arch Gynekol. 1978;13:73.

[7] Stanley JR, Harris AA, Gilbert CF, Lennon YA, Dellinger EH. Magnetic resonance imaging in evaluation of a second trimester ovarian twin pregnancy. Obstet Gynecol. 1994;84:648–51. 9205435

[8] Panda JK. Primary ovarian twin pregnancy. Case report. Br J Obstet Gynecol. 1990;97:540–1. 10.1111/j.1471-0528.1990.tb02527.x 2378832

[9] Bernabei A, Morgante G, Mazzini M, Guerrini E, Fava A, Danero S. Simultaneous ovarian and intrauterine pregnancy: Case report. Am J Obstet Gynecol. 1992;167:134–5. 10.1016/s0002-9378(11)91646-9 1442915

[10] Kalfayan B, Gundersen JH. Ovarian twin pregnancy. Report of a case. Obstet Gynecol. 1963;21:126–8. 13950068

[11] Levin JH, Lacarra M, d’Ablaing G, Grimes DA, Vaermesh M. Mifepristone (RU 486) failure in an ovarian heterotopic pregnancy. Am J Obstet Gynecol. 1990;163:543–4. 10.1016/0002-9378(90)91194-h 2143627

[12] Hallat J. Primary ovarian pregnancy. A report of twenty-five cases. Am J Obstet Gynecol. 1982;143:50–60. 10.1016/0002-9378(82)90683-4 7081312

[13] Phupong V, Ultchaswadi P. Primary ovarian pregnancy. J Med Assoc Thai. 2005;88(4):527–9. 16146259

[14] Raziel A, Golan A, Pansky M, Ron-El R, Bukovsky I, Caspi E. Ovarian pregnancy: a report of twenty cases in one institution Cases Report. Am J Obstet Gynecol. 1990;163:1182–5. 10.1016/0002-9378(90)90685-z 2220925

[15] Herbertsson G, Magnusson S, Benediktsdottir K. Ovarian pregnancy and IUCD use in a defined complete population. Acta Obstet Gynecol Scand. 1987;66:607–10. 10.3109/00016348709022065 3439441

[16] Berger B, Blechner JN. Ovarian pregnancy associated with copper-7 intrauterine device. Obstetr Gynecol. 1978;52:597–600. 724179

[17] Schwartz LB, Carcangiu ML, DeCherney AHX. Primary ovarian pregnancy: a case report. J Reprod Med. 1993;38:155–8. 8445610

[18] Lehfeldt H, Tietze C, Gorstein F. Ovarian pregnancy and the intrauterine device. Am J Obstet Gynecol. 1970;108(7):1005–9. 10.1016/0002-9378(70)90443-6 5479680

[19] Shamma FN, Schwartz LB. Primary ovarian pregnancy successfully treated with methotrexate. Am J Obstet Gynecol. 1992;167:1307–8. 10.1016/s0002-9378(11)91706-2 1442982

[20] Pagidas K, Frishman GN. Nonsurgical management of primary ovarian pregnancy with transvaginal ultrasound-guided local administration of methotrexate. J Minim Invasive Gynecol. 2013;20(2):252–4. 10.1016/j.jmig.2012.10.012 23465263

[21] Di Luigi G, Patacchiola F, La Posta V, Bonitatibus A, Ruggeri G, Carta G. Early ovarian pregnancy diagnosed by ultrasound and successfully treated with multidose methotrexate. A case report. Clin Exp Obstet Gynecol. 2012;39(3):390–3. 23157054

[22] Chelmow D, Gates E, Penzias AS. Laparoscopic diagnosis and methotrexate treatment of an ovarian pregnancy: a case report. Fertil Steril. 1994;62(4):879–81. 10.1016/s0015-0282(16)57020-4 7926103

[23] Annunziata N, Malignino E, Zarcone R. Ovarian pregnancies treated with methotrexate. Panminerva Med. 1996;38(3):190–2. 9009686

